# Cholinergic Agonists and Antagonists Have an Effect on the Metabolism of the Beetle *Tenebrio Molitor*

**DOI:** 10.3390/molecules24010017

**Published:** 2018-12-20

**Authors:** Szymon Chowański, Joanna Pacholska-Bogalska, Grzegorz Rosiński

**Affiliations:** Department of Animal Physiology and Development, Faculty of Biology, Adam Mickiewicz University, 61-712 Poznań, Poland; pacholsk@amu.edu.pl (J.P.-B.); rosin@amu.edu.pl (G.R.)

**Keywords:** insects, muscarinic receptors, insulin-like peptides, carbohydrate metabolism, hemolymph, fat body

## Abstract

Synthetic insecticides are still widely used in plant protection. The main target for their action is the nervous system, in which the cholinergic system plays a vital role. Currently available insecticides have low selectivity and act on the cholinergic systems of invertebrates and vertebrates. Acetylcholine, a cholinergic system neurotransmitter, acts on cells by two types of receptors: nicotinic and muscarinic. In mammals, the role of muscarinic acetylcholine receptors (mAChRs) is quite well-known but in insects, is still not enough. Based on data indicating that the muscarinic cholinergic system strongly affects mammalian metabolism, we investigated if it similarly occurs in insects. We investigated the influence of agonists (acetylcholine, carbachol, and pilocarpine) and antagonists (tropane alkaloids: atropine and scopolamine) of mAChRs on the level of selected metabolites in *Tenebrio molitor* beetle trophic tissues. We analyzed the glycogen content in the fat body and midgut, the total free sugar concentration in the hemolymph and the lipid amount in the fat body. Moreover, we analyzed the levels of insulin-like peptides in the hemolymph. The tested compounds significantly influenced the mentioned parameters. They increased the glycogen content in the fat body and midgut but decreased the concentration of free sugars in the hemolymph. The observed effects were tissue-specific, and were also time- and dose-dependent. We used nonligated and neck-ligated larvae (to eliminate the influence of head factors on tissue metabolism) to determine whether the observed changes are the result of direct or indirect impacts on tissues. The obtained data suggest that the cholinergic system affects the fat body and midgut indirectly and directly and a pleiotropic role for mAChRs exists in the regulation of energy metabolism in insects. Moreover, tested compounds significantly affected the level of insulin-like peptides in hemolymph. Our studies for the first time showed that mAChRs are involved in regulation of insect metabolism of trophic tissues, and act on them directly and indirectly. Improved knowledge about insect cholinergic system may help in searching more selective and environment-friendly solutions in pest management.

## 1. Introduction

Acetylcholine (ACh) is synthesized and distributed in most living organisms, including bacteria, plants, fungi, invertebrates, and vertebrates. This occurrence indicates the crucial role of ACh in cell communication [1]. ACh acts on the animal cells by two types of receptors: nicotinic (nAChRs) and muscarinic (mAChRs). The first type is classified as the ionotropic receptors and the second belongs to the G protein-coupled receptors (GPCRs) [2,3].

Collin et al. [4] showed that insect mAChRs may be divided into two groups. These authors identified and characterized two classes of mAChRs in *Drosophila melanogaster* and *Tribolium castaneum*. The first class, called mAChRs type A (mAChRs-A), shows high structural and pharmacological similarity to mammalian mAChRs. They are very sensitive to agonists (ACh and muscarine) as well as to antagonists (atropine, scopolamine, and 3-quinuclidinyl benzilate) [4]. The second identified class, mAChRs type B (mAChRs-B), possesses significantly different properties when compared with insect mAChRs-A and mammalian mAChRs. The effective concentration (EC_50_) of ACh for the mAChRs-B is similar to the EC_50_ for mAChRs-A; however, mAChRs-B receptors are more than 1000-times less sensitive to muscarine than mAChRs-A. Moreover, these receptors have a very low affinity to antagonists such as atropine, scopolamine, or 3-quinuclidinyl benzilate [4]. The presence of two classes of mAChRs in insects was also confirmed by Ren et al. [5]. Studies conducted by these authors showed that mAChRs-A interact with the G_q/11_ protein, whereas mAChRs-B interact with G_i/o_ proteins. Moreover, Xia et al. [6] reported that in *D. melanogaster* they had identified a third class of mAChRs-type C (mAChRs-C). As it was shown by Collin et al. [4] and Xia et al. [6], the expression of mAChRs in *D. melanogaster* differs strongly according to the developmental stage, sex, and part of body. Moreover, cross-talk between signalization *via* mAChRs and other neurotransmitter systems occurs in insects. Such cross-talk was shown for muscarinic and octopaminergic GPCRs. For example, atropine blocked sustained neuroexcitation evoked by octopamine in the central nervous system of *D. melanogaster* [7], whereas carbachol and oxotremorine affect octopamine stimulated cAMP production in cells of *Periplaneta americana* ventral nerve cords [8].

Knowledge about the participation of mAChRs in the regulation of insect physiology has grown but is still not enough. The available data indicate that these receptors are involved in controlling the flight muscles in *Schistocerca gregaria* [9] and in the regulation of antennae movements in the stick insect *Carausius morosus* [10]. Moreover, muscarinic cholinergic transmission is involved in the regulation of behaviour, memory, and learning in *Apis mellifera*, as has been shown by Cano Lozano and Gauthier [11] and Ismail et al. [12]. Malloy et al. [13] showed that agonists and antagonists of mAChRs influence, in a dose-dependent manner, the heart rate of *D. melanogaster* larvae. Nevertheless, there are no available data about the contribution of the muscarinic system in the regulation of insect metabolism what has been shown in mammals. For example, Gautam et al. [14] indicated that M_3_ mAChRs receptors are involved in the regulation of food intake in mice. The expression of the M_3_ receptors has been observed in almost all organs of the digestive system such as salivary glands, stomach, gut [15], pancreas [14], and liver [16]. Muscarinic receptors take part in the regulation of, e.g., the secretion of saliva, hydrochloric acid, somatostatin, and probably pepsinogen [15]. Vatamaniuk et al. [17] showed that the activation of M_3_ receptors in the hepatocytes increases the synthesis of glucose; and the glycogen content in liver cells.

The mAChRs has been localized in the corpora cardiaca/corpora allata (CC/CA) complex. This structure is engaged in the secretion of various insect neurohormones such as prothoracicotropic hormone in *Bombyx mori* [18] or insulin-like peptides (ILPs)—the crucial insect neuropeptides involved in the regulation of carbohydrate metabolism [19]. The secretion of ILPs from the CC/CA complex is regulated by mAChRs. Shirai et al. [19] demonstrated that carbachol increases the secretion of the ILPs from the CC/CA, whereas atropine causes an inhibition of this process. Moreover, these authors confirmed the expression of mAChRs in the cells synthesizing and secreting ILPs in *B. mori*. Insect ILPs are structurally and functionally analogous to vertebrate insulin, insulin-like growth factors (IGFs), and relaxin [20]. The ILP genes encode precursors similar to vertebrate preproinsulin, which consist of signal peptide, B-chain, C-chain, and A-chain, starting from the *N*-terminus [21]. The mature ILPs, similarly to human insulin, consist of an A and B chain linked with disulfide bonds. The number and position of the cysteine residues are highly conserved [21,22]. Similarly, in the *T. castaneum* beetle, all four ILPs contain both A and B chains [23] and reveal basic folding similarities to mammalian insulin [24]. The DILP5 from *D. melanogaster* was also proven to have properties very similar to mammalian insulin because the synthetic DILP5 binds and activates the human insulin receptor and lowers the glucose level in rats [24]. Moreover, Sevala et al. [25], using anti-bovine insulin antibodies, localized ILPs in *T. molitor* brain in all developmental stages. Similar results were obtained by Teller et al. [26]. These authors confirmed the presence of ILPs in *T. molitor* hemolymph, brain, and midgut.

The aim of our study was to determine whether applications of the agonists (ACh, carbachol, and pilocarpine) and antagonists (atropine and scopolamine) affect the metabolism of carbohydrates and lipids in the beetle, *T. molitor*. Based on the data that carbachol and atropine affect the secretion of ILPs, we supposed that the agonist and antagonists of the mAChRs may have effects on carbohydrates levels in the hemolymph or glycogen content of the trophic tissues in an indirect way. Due to the above, we also decided to check the level of the ILPs in the beetle hemolymph.

## 2. Results

### 2.1. Changes in Glycogen Content in the Fat Body

**Figure 1:**
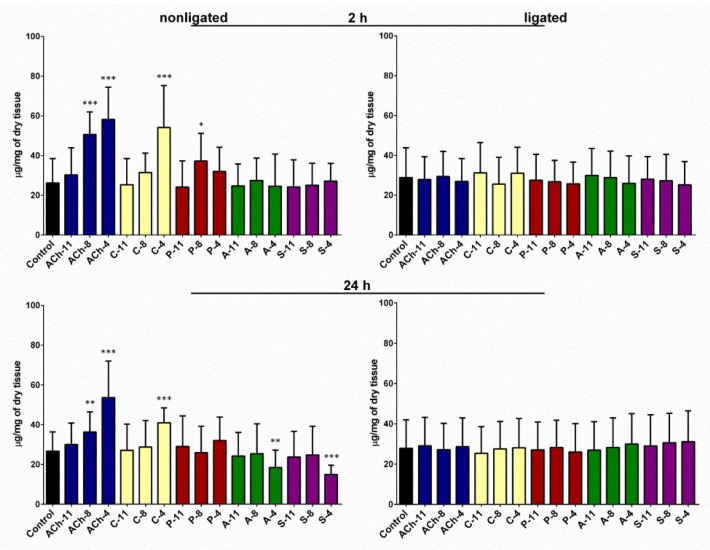
Glycogen content in the fat body of nonligated and neck-ligated larvae of *T. molitor* beetles injected with acetylcholine (ACh), carbachol (C), pilocarpine (P), atropine (A), and scopolamine (S) at concentrations of 10^−11^ (X-11), 10^−8^ (X-8) and 10^−4^ M (X-4) for each compound. The samples were isolated 2 or 24 h after injection. The control larvae were treated with saline. Mean values ± SD are given for 15–25 individuals. Statistically significant differences from the control values are indicated by asterisks (Student’s *t*-test), where * *p* ≤ 0.05, ** *p* ≤ 0.01, and *** *p* ≤ 0.001.

Injection of the tested agonists into the nonligated larvae caused changes in the amount of glycogen in the fat body after 2 and 24 h (one-way ANOVA; for 2 h: F = 14.17; *df* = 169; *p* < 0.0001; for 24 h: F = 7.675; *df* = 167; *p* < 0.0001) (Figure 1). The strongest effect was noted for ACh. Injection of 2 µL of this agonist at 10^−4^ M increased the glycogen content in the fat body by 120% after 2 h of incubation (Student’s *t*-test; *t* = 6.446; *df* = 31; *p* < 0.0001). Similar effects were also observed for lower concentrations of ACh but they were weaker (increase by 92% for 10^−8^ M and 15% for 10^−11^ M). Carbachol, similarly to ACh, induced dose-dependent changes in the analyzed parameter with the highest response at 10^−4^ M (Student’s *t*-test; *t* = 4.805; *df* = 33; *p* < 0.0001). The impact of pilocarpine was weaker but still significant. The effects caused by ACh and carbachol was prolonged and lasted for 24 h. At a concentration of 10^−4^ M, ACh and carbachol increased glycogen level from 26.7 (±9.6) up to 53.6 (±18.3) and up to 40.9 (±7.5) µg/mg of dry tissue, respectively. The opposite effect was noted in insects treated with antagonists but only in the group after 24 h of incubation (one-way ANOVA; for 2 h: F = 0.2527; *df* = 110; *p* = 0.9376; for 24 h: F = 2.2120; *df* = 137; *p* = 0.0458). Atropine decreased the glycogen amount in the tissue by 30% and the scopolamine by 44%. In both cases, we noticed the highest effect at a concentration of 10^−4^ M.

In experiments carried out on the ligated larvae, we did not observe significant changes in the glycogen content in the fat body tissue after application of any compounds tested in both time variants of incubation (one-way ANOVA; for 2 h: F = 0.3896; *df* = 272; *p* = 0.9811; for 24 h: F = 0.2210; *df* = 283; *p* = 0.9992).

### 2.2. Changes in Glycogen Content in the Midgut

**Figure 2:**
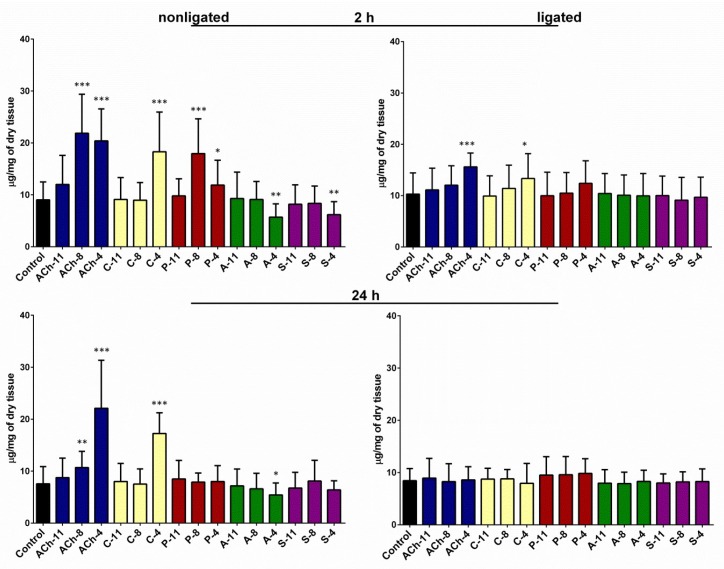
Glycogen content in the midgut of nonligated and neck-ligated larvae of *T. molitor* beetles injected with acetylcholine (ACh), carbachol (C), pilocarpine (P), atropine (A), and scopolamine (S) at concentrations of 10^−11^ (X-11), 10^−8^ (X-8) and 10^−4^ M (X-4) for each compound. The samples were isolated 2 or 24 h after injection. The control larvae were treated with saline. Mean ± SD are given for 15–25 individuals. Statistically significant differences from the control values are indicated by asterisks (Student’s *t*-test), where * *p* ≤ 0.05, ** *p* ≤ 0.01, and *** *p* ≤ 0.001.

In control insects injected with saline, the average level of glycogen in the midgut was 9.04 (±3.45) and 7.58 (±3.29) µg/mg of dry tissue after 2 and 24 h of incubation, respectively. We did not observe significant differences between the ligated and nonligated larvae for this parameter. The glycogen content in the midgut tissue was significantly higher in the insects injected with agonists than in the control group for both incubation times (one-way ANOVA; for 2 h: F = 15.32; *df* = 169; *p* < 0.0001; for 24 h: F = 22.64; *df* = 159; *p* < 0.0001) (Figure 2). At the highest concentration tested, the ACh increased the level of glycogen up to 20.39 (±6.16) and up to 22.10 (±9.24) µg/mg of dry tissue after 2 and 24 h of incubation, respectively. The application of carbachol caused a significant increase in the amount of glycogen in the midgut tissue only at 10^−4^ M, up to 18.29 (±7.65) and 17.24 (±3.99) µg/mg of dry tissue after 2 and 24 h, respectively. Pilocarpine evoked an increase in the level of glycogen in the midgut only in variants with 2 h of incubation. The highest level of glycogen (17.93 (±6.69) µg/mg of dry tissues) was noted after the application of pilocarpine at a concentration of 10^−8^ M.

Antagonists reduced the glycogen level in the midgut for both incubation times. Atropine at a concentration of 10^−4^ M reduced the amount of glycogen in the midgut down to 5.71 (±2.55) and 5.41 (±2.30) µg/mg of dry tissue after 2 and 24 h of incubation, respectively, whereas scopolamine caused significant decline in this parameter in only 2 h—down to 6.18 (±2.49) µg/mg.

We also observed changes in the glycogen content in the midgut of neck-ligated larvae but only in the case of ACh and carbachol. Injection of these two agonists at a concentration of 10^−4^ M increased the content of the glycogen in the midgut after 2 h of incubation by 53% and 30%, respectively (Student’s *t*-test; for Ach: *t* = 5.352; *df* = 47; *p* < 0.0001; for carbachol: *t* = 2.188; *df* = 40; *p* = 0.0346). Pilocarpine also caused an increase in the glycogen content in the neck-ligated larvae but the changes were not statistically significant. Neither of the antagonists tested influenced the glycogen content of the midgut in the neck-ligated larvae.

### 2.3. Changes in the Total Free Sugars Concentration in the Hemolymph

**Figure 3:**
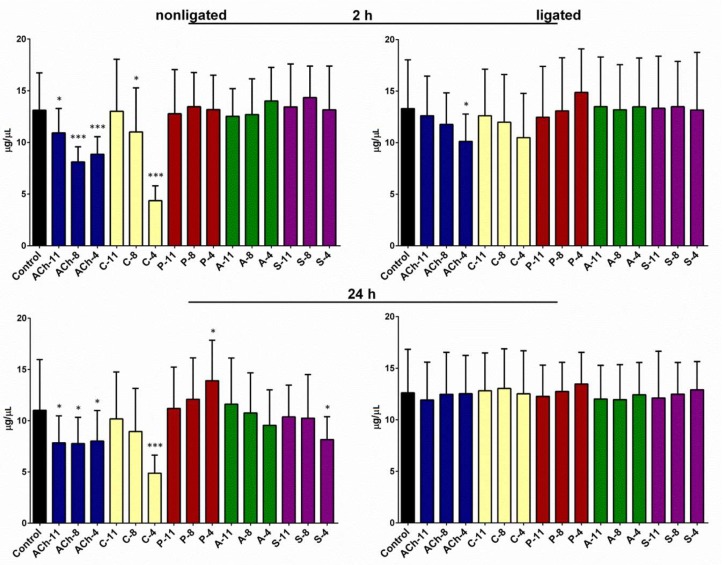
Free sugars concentration in the hemolymph of nonligated and neck-ligated larvae of *T. molitor* beetles injected with acetylcholine (ACh), carbachol (C), pilocarpine (P), atropine (A), and scopolamine (S) at concentrations of 10^−11^ (X-11), 10^−8^ (X-8), and 10^−4^ M (X-4) for each compound. The samples were isolated after 2 and 24 h of incubation. The control larvae were treated with saline. Mean values ± SD are given from at least 15 determinations. Statistically significant differences of the test values from the control values are indicated by asterisks (Student’s *t*-test), where * *p* ≤ 0.05 and *** *p* ≤ 0.001.

For insects injected with saline, the average concentration of the total sugars in the hemolymph was 13.12 (±3.61) and 11.01 (±4.95) µg/µL after 2 and 24 h of incubation, respectively. In the ligated larvae, we noticed only a slight increase in the concentration of sugars in the hemolymph. All tested compounds induced changes in the total sugar level in the hemolymph of the ligated insects (one-way ANOVA; for 2 h: F = 9.873; *df* = 323; *p* < 0.0001; for 24 h: F = 6.871; *df* = 292; *p* < 0.0001) (Figure 3). The hypoglycemic effect was observed in the hemolymph of the larvae injected with ACh at each concentration tested after the 2 and 24 h incubations. At 10^−4^ M ACh, the average concentration of total sugars in the hemolymph was approximately 33% and 28% lower than in the control, after 2 h and 24 h incubation, respectively. The same tendency was observed after the application of carbachol at 10^−4^ M, which lowered the sugar concentration in the hemolymph to 4.36 (±1.44) and 4.87 (±1.77) µg/µL, after 2 h and 24 h incubation, respectively. The third tested agonist, pilocarpine, at a concentration of 10^−4^ M, caused a hyperglycemic effect. It increased the sugar level in the hemolymph after 24 h incubation, and the average level of total sugars was 26% higher than that in the control insects. Changes in the analyzed parameter after application of the antagonist were observed only after the long-term incubation (Figure 3). For both antagonists, a hypoglycemic effect was noted.

In experiments conducted on the ligated larvae, significant changes in total sugars concentration were noted after 2 h from application of the ACh at a concentration of 10^−4^ M. Carbachol and pilocarpine also induced slight effects after 2 h: hypo- and hyperglycemia, respectively. We did not observe any changes in analyzed parameter in the neck-ligated larvae after 24 h of incubation (Figure 3).

### 2.4. Changes in the Lipids Contents in the Fat Body

**Figure 4:**
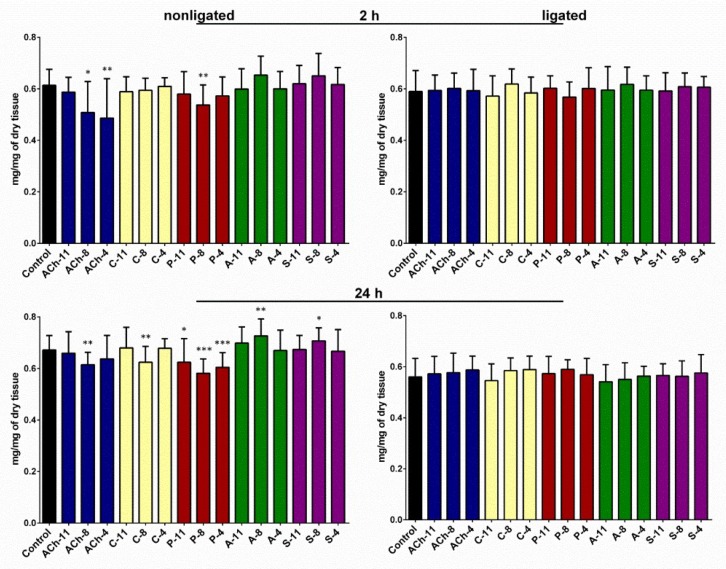
Total lipid content in the fat body of nonligated and neck-ligated larvae of *T. molitor* beetles injected with acetylcholine (ACh), carbachol (C), pilocarpine (P), atropine (A), and scopolamine (S) at concentrations of 10^−11^ (X-11), 10^−8^ (X-8), and 10^−4^ (X-4) M, respectively, for each compound. The samples were collected after 2 and 24 h of incubation. The control larvae were treated with saline. Mean ± SD are given from at least 15 determinations. Statistically significant differences from the control values are indicated by asterisks (Student’s *t*-test), where * *p* ≤ 0.05, ** *p* ≤ 0.01, and *** *p* ≤ 0.001.

The average lipids content in the fat body tissue collected from control larvae was 0.61 (±0.06) and 0.67 (±0.06) mg/mg of dry tissue 2 and 24 h from injection, respectively. We noticed that the lipid level in ligated insects was lower but the changes were statistically insignificant. The total amount of lipids in the fat body was strongly affected by the application of the tested compounds in both time variants of the nonligated larvae (one-way ANOVA; for 2 h: F = 4.371; *df* = 271; *p* < 0.0001; for 24 h: F = 7.579; *df* = 305; *p* < 0.0001) (Figure 4). All used agonists significantly reduced the lipid content in the fat body. The effects were especially strong after 24 h time variant in case of ACh and pilocarpine (Figure 4). For both agonists, the strongest effect was observed at a concentration of 10^−8^ M. ACh evoked decrease in the average lipids content in the fat body by 17% and 10% and pilocarpine 13% and 14% in the 2 and 24 h time variants, respectively. Carbachol affected this parameter only after 24 h of incubation.

The total amount of lipids in the fat body isolated from insects treated with atropine and scopolamine was higher than in the control insects. Nevertheless, the significant changes were observed only in the 24 h time variant after the application of these compounds at a concentration of 10^−8^ M, which increased the lipid content up to 0.73 (±0.07) and 0.71 (±0.05) mg/mg of dry tissue in the case of treatments with atropine and scopolamine, respectively. We did not observe changes in the lipids content in the fat body isolated from the neck-ligated larvae. In the case of all compounds tested, the lipid content was maintained at a similar level.

### 2.5. Level of Insulin-Like Peptides in the Hemolymph

**Figure 5:**
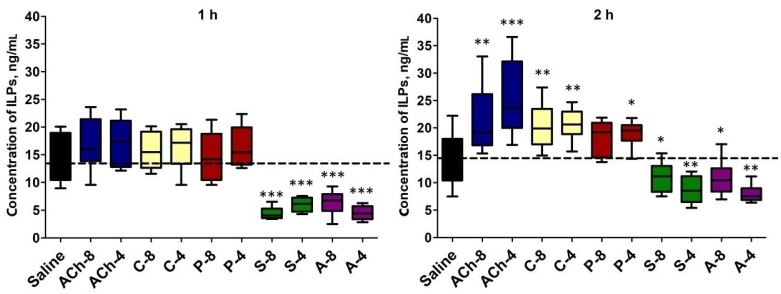
Concentration of ILPs (ng/mL) in the hemolymph of *T. molitor* larvae injected with acetylcholine (ACh), carbachol (C), pilocarpine (P), atropine (A), and scopolamine (S) at concentrations of 10^−8^ (X-8) and 10^−4^ (X-4) M, respectively, for each compound. The samples of hemolymph were collected after 1 and 2 h of incubation. The control larvae were treated with saline. The data are shown as the mean ± SD from 10 to 14 repetitions. Statistically significant differences from the control values are indicated by asterisks (Student’s *t*-test), where * *p* ≤ 0.05, ** *p* ≤ 0.01, and *** *p* ≤ 0.001.

The average level of ILPs in the hemolymph of control, nonligated insects (injected with saline) was 0.62 (±0.19) and 0.63 (±0.20) ng/mL at 1 and 2 h after injection, respectively. Tested compounds affected the level of these peptide neurohormones in both time variants (one-way ANOVA; for 1 h: F = 22.53; *df* = 84; *p* < 0.0001; for 2 h: F = 22.47; *df* = 103; *p* < 0.0001) (Figure 5). The observed response was time-dependent. Antagonists evoked stronger changes after 1 h from injection. In that case, they decreased the level of the ILPs in the hemolymph by more than 2 times, whereas after 2 h of incubation, the level decreased by 25–45% compared to the control. The agonists induced an increase in ILP concentration in the hemolymph but only 2 h after injection (Figure 5). The strongest effect was observed for ACh at a concentration of 10^−4^ M.

## 3. Discussion

In this study, our aim was to determine whether the agonists and antagonists of mAChRs affect insect metabolism. For that, we analyzed metabolic parameters, such as the level of glycogen and lipids in the trophic tissues, the concentration of total free carbohydrates in the hemolymph, and the level of ILPs in hemolymph. We treated larvae of *T. molitor* with three agonists: ACh and carbachol, which affect both the nAChRs and mAChRs, and pilocarpine that acts only on the mAChRs. Moreover, we used two antagonists of the mAChRs: atropine and scopolamine. To get a better understanding of the observed effects, we used neck-ligated larvae to exclude brains factors, similarly to that described by Słocińska et al. [27].

The experiments showed that application of ACh or carbachol caused significant changes in the glycogen level in the midgut and the fat body. The changes were dose-dependent, and the glycogen level increased with the given dose. The same response was observed after the application of pilocarpine (a nonselective agonist of muscarinic receptors), whereas the application of the muscarinic antagonists (atropine and scopolamine) decreased the glycogen content in both tissues. These results may indicate that evoked changes are a result of the muscarinic system modulation. The increase of glycogen content in the fat body and midgut after ACh and carbachol application are coupled with a decrease in the total sugar concentration in the hemolymph. These three tissues cooperate in the regulation of insect metabolism. The fat body is a dynamic tissue involved in multiple metabolic functions, hemolymph plays a role in the distribution of the nutrients around the body, and the midgut is a place of nutrient absorption from the gut lumen. Thus, all mentioned tissues act together to keep the balance of the biochemical parameters [28,29]. Interestingly, pilocarpine induces an increase of in the total sugar concentration in the hemolymph; moreover, the response is delayed, and significant changes are noted 24 h after injection. In contrast to pilocarpine, atropine and scopolamine have an opposite effect on the sugars concentration in the hemolymph. It should be emphasized that ACh and carbachol activate both nicotinic and muscarinic receptors, whereas pilocarpine, atropine, and scopolamine affect only muscarinic receptors. The agonists tested caused an increase in the glycogen content in the fat body and midgut, and at the same time they induced a decrease in the amount of total lipids in these tissues. Data suggest that agonists may activate glycogenesis and lipolysis and/or increase absorption of glucose/trehalose from the hemolymph. The time-dependent differences may be a result of the varying sensitivity of insect mAChRs-A and mAChRs-B to the tested compounds, which was confirmed by Collin et al. [4], and occurs because of differences in the presence of various receptors types in the tissues or can be result of indirect effects.

Obtained data showed that insect mAChRs are involved in regulation of metabolism; in agreement with the data for vertebrates. In this group, cholinergic signaling by the mAChRs plays a significant role in the regulation of metabolism. In mammals, mAChRs are present in the salivary glands, stomach, intestine [15], liver [16], and pancreas [30]. Gautam et al. [31] showed that mice with knockdown of M_3_ mAChRs in cells of the pancreatic *β*-islets demonstrated a high intolerance for glucose, whereas overexpression of M_3_ mAChRs caused an increased production of insulin. Moreover, Vatamaniuk et al. [17] indicated that the activation of M_3_ mAChRs in hepatocytes induced enhanced glucose synthesis and glycogen content in liver cells.

To exclude that observed changes of metabolic parameters of fat body are result of influence of tested compounds on secretion of neurohormonal agents by the corpora cardiaca (CC) we applied a simple ligature bioassay system described by Kopeć [32]. The larvae of *T. molitor* beetles were ligated around the neck to eliminate the influence of head factors on the fat body metabolism. The experiment showed that the tested compounds did not affect the fat body in the ligated larvae. Likely, the changes observed in the fat body are a result of indirect effects because the mAChRs have not been identified in fat body so far. Metabolic activity of this tissue undergoes complex regulation by numerous neurohormonal agents. Two main factors are adipokinetic hormone secreted by CC [33] and insulin-like peptides released from CC [34]. Studies carried out by Satake et al. [32] showed that in *Bombyx mori* ILPs (called as bombyxins in this species) are involved in the regulation of carbohydrate metabolism. Injection of these neurohormones induced a strong, dose-dependent hypoglycemia and increased the activity of trehalase in the midgut and muscles. Thus, hypoglycemia may be a result of the increased hydrolysis of trehalose, which enhances the intake of glucose by muscles and midgut. On the other hand, Satake et al. [34] showed that ILPs decrease the glycogen content in the fat body, which does not correspond with our result. However, the effect of insulin on the glycogen accumulation in the fat body of *B. mori* larvae also has been shown to depend on the larval growth rate. When larvae were in the active growth period, before reaching the critical weight for metamorphosis, the insulin increased the accumulation of glycogen, whereas in larvae at the terminal growth period, the insulin treatment resulted in an increased mobilization of glycogen [35]. Brown et al. [36] also showed that in *Aedes aegypti* the ILP3 elevated glycogen and lipids stores in the decapitated females to the same levels as the controls 24 h after the injection.

Importantly, Shirai et al. [19] demonstrated that muscarinic receptors are involved in secretion of the ILPs. Carbachol increased the secretion of the ILPs from the CC, whereas atropine and scopolamine inhibited this process. Moreover, this group of researchers showed that muscarinic receptors are expressed by cells producing ILPs. Because of the aforementioned, we analyzed the ILP levels in the hemolymph. Our results are similar to those obtained by Shirai et al. [19], with the level of ILPs increasing after treating the insects with the mAChRs agonists and with the ILPs decreasing after application of the antagonists. ILPs play a key role in determining the growth rate and size, metabolic traits, various forms of stress resistance and fecundity. Thus, the observed changes in the ILP level in the hemolymph of *T. molitor* may be correlated with the changes in the biochemical compound content in the fat body, midgut, and hemolymph. We conducted *in vivo* experiments, so the observed changes of glycogen, lipids, and free sugar content are probably the result of direct and or indirect effects of the tested substances on the tissues. Our results show that disconnecting the head from the rest of the body alters the activity of the muscarinic receptors agonists and antagonists on the fat body, which suggests an indirect effect on this tissue by the tested compounds. On the other hand, the midgut was still sensitive to the used compounds even if the head factors were excluded.

## 4. Materials and Methods

### 4.1. Insects

A stock culture of *T. molitor* L. was maintained at the Department of Animal Physiology and Development, Adam Mickiewicz University in Poznań, as described previously by Rosiński et al. [37]. Beetles were reared under laboratory conditions at 26 °C and approximately 65% relative humidity under a 12 h light/12 h dark cycle, with oatmeal and fresh carrot being provided as food. Only larvae of approximately 110–140 mg were used for the experiments. During the experiments, the insects were kept in a plastic box with a diameter of 10 cm and a height of 12 cm, at a density of 15 individuals per box.

### 4.2. Tested Compounds

Tested agonists (acetylcholine—purity 99%, carbachol—purity 98%, and pilocarpine—purity 98%) and antagonists (scopolamine—purity 98% and atropine—purity 99%) were purchased as hydrochloride salts from Sigma-Aldrich (St. Louis, MO, USA). The chemicals were freshly dissolved on the day of the experiments in saline (274 mM NaCl, 19 mM KCl, 9 mM CaCl_2_, pH 7.0), and the acquired stocks were used to prepare the diluted solutions. Tested compounds were applied by injection with a Hamilton syringe (Hamilton, Reno, NV, USA) through the ventral membrane, between the thorax and abdomen. The insects were injected with 2 µL of solution of tested substances at a concentration of 10^−11^, 10^−8^, and 10^−4^ M to obtain a final concentration in the insect hemolymph equaling 10^−12^, 10^−9^, and 10^−5^ M. After injection, the insects were incubated for 2 or 24 h. Experiments were carried out in two variants: with and without ligature of the neck. The ligation of the head was used to exclude head factors that influence the fat body and gut metabolism. The ligation was made before injection with cotton thread between the head and first thorax segment according to methods described by Słocińska et al. [27]. The wet thread was tied behind the head and thorax. Next, the larvae were left undisturbed for 20 min to allow the determination of any leaking of hemolymph. Then, the larvae were injected with the tested compounds.

### 4.3. Determination of Glycogen Content in Tissues

Glycogen isolation was carried out according to Van Handel’s methods [38], with the changes described previously by Chowański et al. [39]. Tissues isolated from the fat body and midgut were washed with saline to remove hemolymph residues and midgut contents. Next, the samples were placed in Eppendorf tubes, dried to a stable weight at 60 °C under a vacuum (−0.9 atm), and finally, the dry mass was measured. In the next step, samples were lysed in 400 µL of 30% KOH (15 min at 90 °C). Following tissue lysis, a saturated solution of Na_2_SO_4_ and 70% ethanol (1:16; *v/v*) was added to precipitate the glycogen. Next, the samples were centrifuged (centrifuge 2K15, Sigma, Osterode, Germany) at 10,000× *g* for 10 min. The supernatant was removed, and the pellet was washed twice with 800 µL of 96% ethanol. Obtained samples (pellet in 96% ethanol) were stored at −20 °C. Before the glycogen was measured, the ethanol was removed, and the pellet was dissolved in water and shaken for 10 min at 80 °C. The glycogen content in the obtained solution was measured using the colorimetric phenol–sulfuric acid method of Dubois et al. [40] with a Biospectrometer (Eppendorf, Hamburg, Germany). Oyster glycogen (Sigma-Aldrich, USA) was used as a standard.

### 4.4. Measurement of Free Sugars Level in Hemolymph

Total free carbohydrates content in hemolymph was determined by the colorimetric phenol–sulfuric acid method described by Dubois et al. [40]. A sample of 2 µL of hemolymph was collected from the first pair of legs after cutting the tarsus and adding it to 500 µL of 70% ethanol. The samples were left for 24 h to allow the full extraction of the free sugars. Then, the samples were centrifuged (10,000× *g* for 10 min), and the concentration of free carbohydrates was determined with a Biospectrometer. Trehalose (Merck, Kenilworth, NJ, USA) was used as the standard.

### 4.5. Evaluation of the Total Lipids Content in the Fat Body

Lipids isolation was carried out according to Folch et al. [41], with the changes described previously by Chowański et al. [42]. Samples of 5–10 mg of fat body tissue washed with saline were collected in Eppendorf tubes, dried to a stable weight at 60 °C under a vacuum (−0.9 atm), and weighed to obtain the dry weight of the sample. The dried tissue was homogenized in a mixture of chloroform and methanol (2:1, *v/v*) for 2 min and centrifuged at 10,000× *g* for 10 min. The supernatant was transferred to a new tube, and the pellet was washed two times with 100 µL of chloroform–methanol mixture. The collected supernatant was washed three times with 220 µL of 0.29% NaCl. Finally, the solvent was evaporated at 30 °C under a vacuum (−0.9 atm), and the pellet was dissolved again in 1 mL of chloroform–methanol mixture (2:1, *v/v*). Aliquots of the mixture were taken, and the lipid content was measured gravimetrically after the evaporation of the solvent from the samples.

### 4.6. Immunoenzymatic Determination of the Insulin-Like Peptides Level in Hemolymph

An ELISA Insulin Kit was used (DRG Instruments GmbH, Marburg, Germany. Cat.# EIA2935) to determine the level of the insulin-like peptides. Samples of hemolymph were collected from the insects 1 or 2 h after injection of the tested compound. Hemolymph from 8 larvae (4 µL from each larva) was combined in 3 µL of 10% EDTA to obtain 35 µL of final sample. The samples were kept on ice until use. Next, the samples were shaken for 15 min (1400 rpm at 4 °C) on a thermomixer (Eppendorf, Hamburg, Germany) and stored at −20 °C. Before being measured, the samples were centrifuged at 10,000× *g* at 4 °C for 10 min. Analysis was conducted using an ELISA Insulin Kit according to the manual provided by the producer. Samples (25 μL) were added to the wells and incubated with Enzyme Conjugate for 30 min. Next, after removal of the solution, the wells were washed 3 times with wash buffer, and Enzyme Complex was added. After 30 min of incubation and triple washing, the substrate solution was added, and the samples were incubated for 15 min. Finally, the reaction was stopped with the stop solution, and the absorbance was measured (λ = 450 nm) with a BioTek Spectrophotometer (Synergy H1, Winooski, VT, USA). The concentration of the ILPs was calculated based on a standard curve.

### 4.7. Statistics

All data are presented as the mean values ± SD of n number of replicates. For statistical comparison between groups with normal distribution, a one-way ANOVA was used. In addition, for comparison between the control and test group, Student’s *t*-test was used. The normality of the distribution was analyzed with the Shapiro–Wilk test. The statistical analyses were performed using Graph Pad Prism (version 5) software (GraphPad Software Inc, Version 5.01, MacKiev, La Jolla, CA, USA, 1992–2007) (Department of Animal Physiology and Development AMU license). Differences were considered statistically significant at * *p* ≤ 0.05, ** *p* ≤ 0.01, and *** *p* ≤ 0.001.

## 5. Conclusions

Our experiments showed, for the first time, that the muscarinic cholinergic system might be involved in the regulation of insect metabolism. Nevertheless, participation of nicotinic cholinergic system cannot be excluded, especially in the cases of ACh and carbachol. The comparison of data obtained in the experiments conducted on ligated and nonligated larvae showed that the impact of the agonist and the antagonist of the muscarinic receptors on the fat body are probably both indirect and direct. Moreover, the activity of the tested agonist and antagonist are tissue-specific and are significantly time-dependent. Furthermore, we showed that application of muscarinic receptor agonists and antagonists significantly affects the level of insulin-like peptides in hemolymph. It suggests that muscarinic cholinergic system plays a crucial role in regulation of synthesis and/or releasing of these, one of the most important, insect neuropeptides involved in metabolism regulation.
